# Supplementary material for: Stakeholder considerations on acceptability and implementation of a novel rapid test for acute HIV infection: A qualitative study in Indiana
Source: PLOS Glob Public Health. 2025 Oct 30;5(10):e0005366. doi: 10.1371/journal.pgph.0005366 (PMC12574885; doi:10.1371/journal.pgph.0005366)
Supplement: S1 Table — (DOCX) [file pgph.0005366.s001.docx]

S1 Table. Interview Questionnaires

| Staff Interview Questions | Client Interview Questions | |
| --- | --- | --- |
| Can you tell me about the people that you serve?   - About how many clients does your organization serve? - What are some of the typical demographics of the communities you serve?   What are some of the key barriers to accessing HIV testing and treatment that you typically see among your clients (or before they become your clients)?  How do you test people for initial HIV diagnosis?   - What specific test do you use? - How do you feel about the test? Any issues? - What kind of sample do you take? *(Saliva? Blood? Fingerprick?)* - Where do you test them? - Who performs the test? - How often do you test people? *(just see them once and never again? Or do you see them frequently and offer testing often?)* - In an ideal world, how often should we test people? PWID specifically?   What are your thoughts on self-testing? Peer-based testing? CHW-led testing?   - What possible problems could these solve? - What possible problems could these cause?   What about for PWID? Which testing modality do you think would work best?  Do you think testing your clients/letting them know their HIV status changes their health behaviors in any way?  Engineers are currently working on a rapid HIV test that could detect acute HIV infection at least a month earlier than existing tests, but it takes longer (around 60 minutes)   - - Do you think this trade-off would be worthwhile to clients or providers?   - Do you think there is a need to be able to detect HIV earlier for PWID/other at-risk populations? | Have you ever been tested for HIV?   - When was the last time? - Why did you decide to get tested? - How often do you get tested?   - How often would you like to get tested ideally? - What was the test like?   - Who did the test? (Doctor? CHW? Peer?)   - Finger-prick? Saliva?   - How long did it take to know results?   - Any follow-up?   Is it easy to access HIV testing whenever you want it?  What are the barriers?  Have you ever heard of an HIV rapid test?   - In your opinion, who should perform the test? (probe: chw, nurse, doctor, shelter staff, needle exchange service staff) - Where would you ideally want to take the test? (probe: street, shelter, site, other) - Would you be willing to conduct your own test? Or prefer someone you know to do it for you?   Have you ever heard of a peer recovery coach/certified recovery specialist/community health worker?   - Would you like to be able to work with one? - If yes: how might they be helpful to you? - If no: why not?   If a peer recovery coach had HIV tests on them and could test you anytime, would that be useful to you? Would you seek them out for HIV testing? Why/why not?  If you could test yourself for HIV, with a test kind of like a pregnancy test that you could get at any pharmacy… would that be useful to you? Would you like the ability to be able to test yourself anytime? Why/Why not?   - What are some benefits of being able to test yourself? - What do you think are possible downsides? - When or how often would you test yourself? - Would your behaviors change if you knew your were positive/negative? - Would you know what to do/where to go/who to call if you were positive?   Engineers are currently working on a rapid HIV test that could detect HIV infection at least a month earlier than existing tests, but it would take longer (around 1hr instead of 15 minutes)   - - Do you think there is even a need to be able to detect HIV earlier?   - Do you think this trade-off (takes longer but get results earlier) would be worthwhile to you? Why/why not? | *Demographics:*  What is your gender identity?    What is your age?  What is your race/ethnicity?  Are you currently experiencing homelessness?  Have you ever injected drugs?   - If yes, how many times did you inject drugs in the past month? - Of the times that you injected drugs, what proportion of those times did you share a needle or syringe?   How many sexual partners have you had in the past year?   - Do you know your partner(s) HIV status? - Have you ever had an HIV-positive sex partner? - What proportion of the time did you or your partner(s) use a condom while engaging in sexual activity? (never, rarely, sometimes, half, most, always/almost always) |
