# Supplementary material for: Stakeholder considerations on acceptability and implementation of a novel rapid test for acute HIV infection: A qualitative study in Indiana
Source: PLOS Glob Public Health. 2025 Oct 30;5(10):e0005366. doi: 10.1371/journal.pgph.0005366 (PMC12574885; doi:10.1371/journal.pgph.0005366)
Supplement: Attachment — Submitted filename: comments.docx [file pgph.0005366.s003.docx]

I acknowledge the efforts of the authors in examining the acceptability and implementation considerations for a novel rapid test designed to detect acute HIV infection (AHI). The study addresses a critical gap in HIV testing by focusing on AHI detection, a period of high transmissibility. It aligns with the "Ending the HIV Epidemic" initiative. Unfortunately, several limitations are noted demanding major revisions.

Some of these are highlighted below;

- The claim that "none can accurately detect HIV in the early AHI phase" is too broad and doesn't reflect the capabilities of lab-based NAT and fourth-generation assays. The authors should clarify that they are primarily discussing the limitations of rapid, point-of-care tests, not all HIV tests. The importance of rapid AHI tests lies in making early detection more accessible, particularly to high-risk populations who may not regularly visit clinics or have easy access to lab testing.
- The small sample size of clients (n=5) raises concerns about the generalizability of their views. The authors acknowledge that thematic saturation was not reached among this group.

While the authors mention the challenges in recruiting clients, further efforts to increase the sample size or a more in-depth justification for the limited sample would strengthen the study.

- Recruiting clients *via* flyers posted at HIV organizations may have biased the sample towards those already engaged in testing services.

The authors research should consider more proactive recruitment strategies to reach medically underserved communities not currently engaged in HIV testing or care.

- More detailed demographic information about the staff and client participants should be included. While the client ethnicities are mentioned, the economic background of the clients and the number of years of experience of the staff would be relevant.

Adding this information would help to better understand the context of the data.

- The study is based on data from Indiana, which may limit the generalizability of the findings to other geographic locations or populations with different HIV prevalence rates or healthcare systems.

The authors should acknowledge this limitation and encourage future research in other settings.

- The authors mention the need for a new TPP with detailed specifications for POC AHI testing but do not provide a specific TPP for the MicroRAAD test.

Elaborating on the desired characteristics of the AHI test, such as sensitivity, specificity, and cost, would be beneficial.

- **Inconsistencies:** Line 47, it says "those who test positive to quickly engage in HIV treatment and reduce viral load to untransmissible levels". However, line 56 says, "These tests require a waiting period of up to 90 days after exposure as they cannot detect the virus at the AHI stage". These statements are in conflict with each other and should be addressed in the paper.
