# Supplementary material for: Stakeholder considerations on acceptability and implementation of a novel rapid test for acute HIV infection: A qualitative study in Indiana
Source: PLOS Glob Public Health. 2025 Oct 30;5(10):e0005366. doi: 10.1371/journal.pgph.0005366 (PMC12574885; doi:10.1371/journal.pgph.0005366)
Supplement: Attachment — Submitted filename: Response to Reviewers.docx [file pgph.0005366.s005.docx]

Reviewers' comments:

Reviewer #1: This is a well-written and timely qualitative study that explores stakeholder perspectives on a novel rapid test for acute HIVinfection. The manuscript is grounded in human-centered design and presents meaningful insights for public health implementation, especially among high-risk populations. The methods are appropriate, the findings are clearly presented, and the discussion is thoughtful. With minor revisions to improve clarity in the abstract and introduction, the manuscript will make a strong contribution to the literature on HIVdiagnostics and equity-focused health innovation. However, there are space for improvements.

We thank reviewer 1 for this positive feedback and thoughtful comments to improve our manuscript. Please see responses in blue below.

1. Abstract: Clarify the purpose and novelty of the AHI test earlier, and state the main finding more directly (e.g., "Participants supported earlier detection despite longer wait times").

Thank you for these suggestions – we have revised the abstract accordingly.

2. Introduction: Briefly introduce the AHI test earlier and focus less on technical mechanisms; emphasize its public health significance and relevance for high-risk populations.

We have now added earlier (introduction, 3^rd^ paragraph): *“Novel rapid AHI tests, designed to detect infection earlier and closer to the time of exposure, could transform testing by identifying individuals who are most infectious and linking them to care sooner. Such tests are particularly relevant for high-risk populations, including people who inject drugs, who often face barriers to accessing traditional healthcare.”*

Reviewer #2: This is an important area of research that may reduce the transmission of HIV
The manuscript is well-written, although it contains a few grammatical errors that need correction.
1 The abstract does not have the aim of the study.

We have revised the abstract to more clearly state the aim of the study upfront.

2. Why did the authors like our other stakeholders who made meaningful contributions to the study? These may include opinion leaders, Ministry of Health decision makers, community representatives, etc

3 According to the authors, do they think the stakeholders represented the various sectors and geographical locations?

We have expanded the results and discussion to address these 2 points above, as follows:

Results: “The staff interviewed represented 10 organizations that provide HIV testing services across six counties throughout Indiana (Allen, Marion, Monroe, Scott, Tippecanoe, Vanderburgh), including county health departments, syringe service programs, and healthcare clinics. Some organizations were part of larger networks and partnerships, serving up to 45 counties and partnering with up to 75 other HIV/AIDS organizations.”

Discussion: “The staff we interviewed represented 10 organizations spanning six counties in Indiana, including health departments, syringe service programs, and healthcare clinics. While this diversity captures a range of service models and settings, it may not fully represent all geographic areas of the state or other organizational perspectives. Our study did not include perspectives from broader stakeholders such as policy makers or payers, who could provide important insights into regulatory and funding considerations. Future work must reach broader stakeholders including local health departments and insurance companies to understand implementation considerations related to reporting and reimbursement.”

Reviewer #3:

I acknowledge the efforts of the authors in examining the acceptability and implementation considerations for a novel rapid test designed to detect acute HIV infection (AHI). The study addresses a critical gap in HIV testing by focusing on AHI detection, a period of high transmissibility. It aligns with the "Ending the HIV Epidemic" initiative. Unfortunately, several limitations are noted demanding major revisions.

We thank reviewer 3 for this positive feedback and thoughtful comments to improve our manuscript. Please see responses in **blue** below.

Some of these are highlighted below;

- The claim that "none can accurately detect HIV in the early AHI phase" is too broad and doesn't reflect the capabilities of lab-based NAT and fourth-generation assays. The authors should clarify that they are primarily discussing the limitations of rapid, point-of-care tests, not all HIV tests. The importance of rapid AHI tests lies in making early detection more accessible, particularly to high-risk populations who may not regularly visit clinics or have easy access to lab testing.

We have revised the abstract to clarify this point.

- The small sample size of clients (n=5) raises concerns about the generalizability of their views. The authors acknowledge that thematic saturation was not reached among this group.

While the authors mention the challenges in recruiting clients, further efforts to increase the sample size or a more in-depth justification for the limited sample would strengthen the study.

We have expanded the discussion to emphasize this limitation and justification: “Given the sensitivity of the topic and the vulnerability of this population, our study relied on community partnerships with established client relationships. As such, our sample size of clients was relatively small and thematic saturation was not reached among this participant group. Recruitment was constrained by the ethical need to rely on passive, low-burden strategies during the COVID-19 pandemic. These constraints limited sample size but were necessary to ensure participant safety and confidentiality. Nonetheless, our findings provide important context-specific insights for technology development and offer significant support for future research around AHI diagnostics.”

- Recruiting clients *via* flyers posted at HIV organizations may have biased the sample towards those already engaged in testing services.

The authors research should consider more proactive recruitment strategies to reach medically underserved communities not currently engaged in HIV testing or care.

We expanded on this point in the discussion: “… a key limitation of this study is that recruitment relied on flyers posted at HIV service organizations, which likely biased the client sample toward individuals already engaged in HIV testing or care. As a result, the perspectives of medically underserved or less connected populations may not be fully captured. Future research should adopt proactive recruitment strategies, such as outreach in non-clinical community settings, to reach individuals not currently accessing testing or care.”

- More detailed demographic information about the staff and client participants should be included. While the client ethnicities are mentioned, the economic background of the clients and the number of years of experience of the staff would be relevant.

Adding this information would help to better understand the context of the data.

We have added this information into results, paragraph 1.

- The study is based on data from Indiana, which may limit the generalizability of the findings to other geographic locations or populations with different HIV prevalence rates or healthcare systems.

The authors should acknowledge this limitation and encourage future research in other settings.

We added to the discussion: “Because the study was conducted in Indiana, findings may not generalize to regions with different HIV prevalence rates, healthcare infrastructure, or sociopolitical environments. Further studies in diverse geographic and epidemiologic contexts are needed to validate and expand these findings.”

- The authors mention the need for a new TPP with detailed specifications for POC AHI testing but do not provide a specific TPP for the MicroRAAD test.

Elaborating on the desired characteristics of the AHI test, such as sensitivity, specificity, and cost, would be beneficial.

We have revised the discussion to elaborate on the desired characteristics guiding the design of the microRAAD.

- **Inconsistencies:** Line 47, it says "those who test positive to quickly engage in HIV treatment and reduce viral load to untransmissible levels". However, line 56 says, "These tests require a waiting period of up to 90 days after exposure as they cannot detect the virus at the AHI stage". These statements are in conflict with each other and should be addressed in the paper.

We have revised to clarify that *once* HIV is detected *then* treatment can be initiated, but that current rapid tests have a diagnostic window that misses many acute infections.

Journal Requirements:

1. Please amend your detailed Financial Disclosure statement. This is published with the article. It must therefore be completed in full sentences and contain the exact wording you wish to be published.

i. State the initials, alongside each funding source, of each author to receive each grant.
ii. State what role the funders took in the study. If the funders had no role in your study, please state: “The funders had no role in study design, data collection and analysis, decision to publish, or preparation of the manuscript.”

Addressed

2. Please send a completed 'Competing Interests' statement, including any COIs declared by your co-authors. If you have no competing interests to declare, please state "The authors have declared that no competing interests exist". Otherwise please declare all competing interests beginning with the statement "I have read the journal's policy and the authors of this manuscript have the following competing interests:"

Addressed

3. Please ensure that your Ethics Statement is available in its entirety at the beginning of your Methods section, under a subheading 'Ethics Statement'. It must include:

1) The name(s) of the Institutional Review Board(s) or Ethics Committee(s)

2) The approval number(s), or a statement that approval was granted by the named board(s)

3) (for human participants/donors) - A statement that formal consent was obtained (must state whether verbal/written) OR the reason consent was not obtained (e.g. anonymity).

NOTE: If child participants, the statement must declare that formal consent was obtained from the parent/guardian.

Addressed

4. Please upload separate figure files in .tif or .eps format. Also, remove the figures from your manuscript file but keep the legends.

For more information about figure files please see our guidelines:

<https://journals.plos.org/globalpublichealth/s/figures> 
<https://journals.plos.org/globalpublichealth/s/figures#loc-file-requirements>

Addressed

5. We have noticed that you have uploaded Supporting Information files, but you have not included a list of legends. Please add a full list of legends for your Supporting Information files after the references list.

Addressed

6. In the online submission form, you indicated that “Data, coding schemes, and interview guides are available by request. Please email Natalia Rodriguez at [natalia@purdue.edu](mailto:natalia@purdue.edu).”.

All PLOS journals now require all data underlying the findings described in their manuscript to be freely available to other researchers, either 
1. In a public repository, 
2. Within the manuscript itself, or 
3. Uploaded as supplementary information.

This policy applies to all data except where public deposition would breach compliance with the protocol approved by your research ethics board. If your data cannot be made publicly available for ethical or legal reasons (e.g., public availability would compromise patient privacy), please explain your reasons by return email and your exemption request will be escalated to the editor for approval. Your exemption request will be handled independently and will not hold up the peer review process, but will need to be resolved should your manuscript be accepted for publication. One of the Editorial team will then be in touch if there are any issues.

We have emailed the editor to explain our exemption request.

7. Some material included in your submission may be copyrighted. According to PLOS’s copyright policy, authors who use figures or other material (e.g., graphics, clipart, maps) from another author or copyright holder must demonstrate or obtain permission to publish this material under the Creative Commons Attribution 4.0 International (CC BY 4.0) License used by PLOS journals. Please closely review the details of PLOS’s copyright requirements here: PLOS Licenses and Copyright. If you need to request permissions from a copyright holder, you may use PLOS's Copyright Content Permission form.

Please respond directly to this email or email the journal office and provide any known details concerning your material's license terms and permissions required for reuse, even if you have not yet obtained copyright permissions or are unsure of your material's copyright compatibility.

Potential Copyright Issues:

Figure 1: Please confirm whether you drew the images / clip-art within the figure panels by hand. If you did not draw the images, please provide (a) a link to the source of the images or icons and their license / terms of use; or (b) written permission from the copyright holder to publish the images or icons under our CC-BY 4.0 license. Alternatively, you may replace the images with open source alternatives. See these open source resources you may use to replace images / clip-art:

- <https://commons.wikimedia.org>
- <https://openclipart.org/>

If the reviewer comments include a recommendation to cite specific previously published works, please review and evaluate these publications to determine whether they are relevant and should be cited. There is no requirement to cite these works unless the editor has indicated otherwise.

We have emailed the editor to explain: The figure was created using Canva Pro. All icons and images are sourced from Canva’s licensed library and are used in accordance with [Canva’s Content License Agreement](https://www.canva.com/policies/content-license-agreement/), which permits academic publication under CC-BY licenses.

Additional Editor Comments (if provided):

Thank you for submitting your manuscript to PLOS Global Public Health.

In addition to the reviewers' comments, please address the following points:

1. The title of the article does not reflect the objectives or the design of the study; please adjust.

We have revised the title to: “Stakeholder considerations on acceptability and implementation of a novel rapid test for acute HIV infection: a qualitative study in Indiana”

2. Abstract: The results section is underdeveloped; please expand and condense the Background.

We have revised the abstract completely to address several reviewer comments and have expanded on the results section of the abstract.

3. Methods: Add a short paragraph on study design, cite the COREQ guidelines and attach these as an appendix, making sure all of the items in the checklist are presented in the text.

We have added this to Methods, cited COREQ guidelines, and attached our COREQ checklist as a supplemental file.

4. Methods: Please clarify what types of organizations were invited to participate (HIV organizations is somewhat vague).

We have clarified this in Methods: “Any staff member of an HIV care organization, defined as any organization that provides HIV testing and linkage to care (e.g., clinic, health department, syringe service programs), was eligible to participate if they oversaw or conducted HIV testing (direct users of HIV tests).”

5. Results: Please nuance your statement "Acceptability of the described AHI rapid test was high." - You then state "All participants agreed there was a need to detect HIV earlier in high-risk populations." however this does not directly support your first statement. There is a need to significantly expand what is presented in this section, differentiate it by clients and staff which probably have different perspectives of acceptability, and beyond saying that acceptability is high you should describe the reasons for this. Consequently, I suggest transforming table 1 into narrative text to expand the acceptability section.

We have rewritten the content of the table into narrative text that provides additional nuance, differentiating between clients and staff perspectives, and elaborating on the reasons for acceptability.

6. Results: Table 3 does not add value as it is; please transform it to narrative text to expand the section "Implementation Considerations."

Thank you for this suggestion. We have removed table 3 and replaced it with narrative text that contains the same original quotes from the table. Within the text we made **bold** the key themes we wanted to point out, please let us know if this is not acceptable and we can unbold.

Finally, please clarify whether the senior author holds any commercial interest in the test under evaluation, and expand on the safeguards implemented to ensure the integrity and impartiality of the study findings.
We have revised the competing interests statement and now specify that any potential conflicts of interest are documented in an annual financial conflict of interest report that is reviewed and approved by Purdue University’s Office of Research for Ethics and Compliance.
